# Operant conditioning: a minimal components requirement in artificial spiking neurons designed for bio-inspired robot's controller

**DOI:** 10.3389/fnbot.2014.00021

**Published:** 2014-07-25

**Authors:** André Cyr, Mounir Boukadoum, Frédéric Thériault

**Affiliations:** Computer Science Department, Cognitive and Computer science, Université du Québec à MontréalMontréal, QC, Canada

**Keywords:** operant conditioning, robot, learning, spiking neurons, adaptive behavior, bio-inspired agents

## Abstract

In this paper, we investigate the operant conditioning (OC) learning process within a bio-inspired paradigm, using artificial spiking neural networks (ASNN) to act as robot brain controllers. In biological agents, OC results in behavioral changes learned from the consequences of previous actions, based on progressive prediction adjustment from rewarding or punishing signals. In a neurorobotics context, virtual and physical autonomous robots may benefit from a similar learning skill when facing unknown and unsupervised environments. In this work, we demonstrate that a simple invariant micro-circuit can sustain OC in multiple learning scenarios. The motivation for this new OC implementation model stems from the relatively complex alternatives that have been described in the computational literature and recent advances in neurobiology. Our elementary kernel includes only a few crucial neurons, synaptic links and originally from the integration of habituation and spike-timing dependent plasticity as learning rules. Using several tasks of incremental complexity, our results show that a minimal neural component set is sufficient to realize many OC procedures. Hence, with the proposed OC module, designing learning tasks with an ASNN and a bio-inspired robot context leads to simpler neural architectures for achieving complex behaviors.

## Introduction

Learning is well recognized by the scientific community as a major feature of intelligence. In biological agents, this concept implies behavioral adaptations from experiences, and it is essential for confronting the high variability of the real world. In this context, learning brings real-time flexible solutions that a reactive response mode alone cannot afford. As an evolutionary process during an organism's lifespan, learning allows an anticipation of the near future by doing actions differently. Many description levels of learning exist as partial explanations of the empirical data founded on the phenomenon. Among alternatives, the neurobiological cellular level seems a coherent approach to understand any learning processes deeply. Whether studies on learning refer to natural or artificial agents, extensive research exists in both domains. In artificial intelligence (AI), including the robotic field, learning rules flourish and the direction depends on the contextual paradigm and goals they are utilized for Watkins (1989) and Sutton and Barto (1998).

Among the existing approaches to robot controllers, the bio-inspiration trend (Bekey, 2005; Floreano and Mattiussi, 2008; Pfeifer et al., 2012) suggests that the principles underlying natural intelligence, of which the learning phenomenon, can be understood theoretically and may be used as inspiration to realize the core computation in AI agents. The derived computational models, once embedded in robotic “brains” with sensory inputs and motor outputs, may then emulate natural intelligent behavior. One important candidate for the development of bio-inspired controllers is the artificial neural network (ANN), an abstraction of the natural counterpart. Like real neurons, artificial neural units use stored weights (memory) to perform the integration of one or more inputs at an input node and generate an activation value. The weights are set via a learning strategy and a transmission function uses the integration result to generate the neural output, typically action potentials that feed one or more other neural inputs in the ANN. The ANN has many interesting properties: the ability to process implicit and fragmentary data, high computation parallelism, and the capacities of generalization, discrimination, classification and pattern completion, all possible in a variety of application contexts.

Among several declinations of ANNs, the latest is represented by the artificial spiking neural network (ASNN) sub-family (Maass, 1997; Gerstner and Kistler, 2002). ASNNs distinguish themselves from preceding ANN models in that the underlying algorithm is inherently suited to resolve temporal cognitive problems. Instead of using rate coding to model the individual neural firing, the time stamping used by the ASNN allows timing, synchronization, and asymmetrical temporal correlations (Izhikevich, 2003) between spikes. ASNNs are also well-adapted for real world applications (see Ponulak and Kasiński, 2011, for a survey). In particular, they do not require prior batch training as much as ANNs and their weight adjustment process is typically much faster than the offline gradient descent algorithm that is often used for ANNs (Wilson and Martinez, 2003). For instance, an ASNN with unsupervised learning rules may learn solutions in just one trial from a first correlated paired of spikes, hence exhibiting a critical ability in a real-time context, particularly for organisms with short life spans, which cannot afford a learning mechanism based on slow synaptic adjustments. Finally, a desirable feature that ASNNs share with ANNs is the possibility of add-on learning rules to cope with uncertainty and complexity when handling dynamic information.

In this work, we use a dynamic ASNN adapted for virtual and physical robotic simulations. It relies on a discrete-time leaky integrate-and-fire (LIF) neural model, implemented as a program that uses lookup tables to specify the synaptic and neural properties. A LIF neuron is a mathematical abstraction of the biological neuron that puts emphasis on the integrative and output functions parts. Simple LIF models (Gerstner and Kistler, 2002; Izhikevich, 2003) consider that time-varying post-synaptic potentials (PSP) are the sources of inputs currents to the neuron (positive or negative), with a subsequent dynamic effect on the membrane potential. The process is typically modeled with the following state equation:

$$C\frac{du(t)}{dt}=-\frac{1}{R}u\left(t\right)+\left(i_{0}\left(t\right)+\sum_{n}w_{j}i_{j}\left(t\right)\right)$$

where *u*(*t*) stands for the cell membrane potential, *C* for the membrane capacitance, *R* for its resistance, and *i* represent the input currents coming through synapses (weights) *w*. A spike is generated whenever *u*(*t*) reaches a predetermined (and constant) threshold θ, after which the membrane potential is reset to a resting value θ_0_ for a refractory period *T_r_*. The leaky term $\frac{1}{R}u\left(t\right)$ allows emulation of passive ionic diffusion, a characteristic forcing the membrane potential to come back slowly to its original equilibrium state.

The integration of the input presynaptic spikes to form the input currents could take different curve shapes, but it is generally approximated by an exponential function to represent the membrane potential's ability to reach the firing threshold. Hence, an input current is typically represented by:

$$i\left(t\right)=\int_{0}^{8}s(\tau -t)e^{-\frac{\tau }{\tau_{s}}}$$

where *s*() represents the presynaptic spike train and τ_*s*_ is the integration time constant.

It should be clear that the previous equations are a simplification of the actual organization and neurophysiologic processes of a real neuron. In particular, the spatial structure, the mapping of pre-synaptic to post-synaptic potentials and the role of neurotransmitters are lumped into simple synaptic weights. However, the model is sufficient to account for many useful neural phenomena.

Our LIF neural model includes all the previous elements, but we use lookup tables to represent the dynamics of the various inputs variables, and ASNNs can be built using them thanks to a software called SIMCOG that we developed (Cyr et al., 2009) (see Supplementary material for details and starting parameters). Here, we use it to demonstrate operant conditioning (OC) in a bio-inspired robotic paradigm, when applied in different contexts through an ASNN.

Obviously, reproducing the natural OC learning process must stand on critical, permanent, and tractable neural components. In this respect, we suggest a constant corpus of elements that are common in multiple OC scenarios. First, we establish a simple model of a habituation rule, a non-associative learning process. Habituation is defined by many temporal features (Thompson and Spencer, 1966; Rankin et al., 2009), but the main ones are a decreasing response to persistent stimuli and a recovery part when they cease. It involves more-or-less persistent modifications at the pre-synaptic element. The functional impact of habituation is thought to minimize redundant information, filtering input and enhancing stimuli novelty (Marsland et al., 2005). The justification to include this learning rule in our OC model is to avoid reflex behaviors from constant input (McSweeney et al., 1996), acting similarly to an intrinsic action selection mechanism. To this end, the original ASNN of SIMCOG was recently enhanced with a novel computational model of habituation (Cyr and Boukadoum, 2013), extended for temporal features.

The next step in a neurobiological perspective about realizing an OC model is the implementation of classical conditioning (CC) (Pavlov, 1927). This type of learning allows agents, through association between repetitive pairs of conditioned (neutral) and unconditioned (meaningful) stimuli to eventually produce a conditioned response that is the same as the unconditioned one (Schmajuk, 2010). CC is considered a passive associative learning skill, since agents have no power to intervene in the conditioning process. For instance, the sea-slug Aplysia Californica, one well-known lower neural system animal model, exhibits aversive CC of the gill and siphon withdrawal reflex (Hawkins, 1984; Glanzman, 1995). One plausible biologically cellular mechanism that partially explains event association in CC is the spike-timing dependent plasticity (STDP) process (Bi and Poo, 1998; Markram et al., 2011), which consists mainly of a directional and causal modification of synaptic strength in relation to the precise timing of paired-spikes within a specific temporal window. As such, an STDP computational model of associative learning was also recently proposed in the ASNN of SIMCOG (Cyr and Boukadoum, 2012).

OC also consists in a form of associative learning where agents modify their behaviors by associating stimuli and responses, actively changing their behavior from past acquired experiences, by the motivation of rewards or punishments. Hence, agents alter their normal behaviors from acquired knowledge by “evaluating” the acts they previously made and the current changes. Pioneers (Thorndike, 1911; Skinner, 1938; Hull, 1943) left many researches as legacy, establishing the foundation of the subject. OC is a phenomenon recognized across modalities and developmental phases (Valente et al., 2012) in almost all natural neural species. In particular, the study of the OC mechanism has greatly benefited from invertebrate models such as the sea slug Aplysia (Brembs, 2003). Still, the structural elements and the precise dynamics involved in the process remain to be determined. As a starting point in the quest for a minimal components requirement defining OC, the present literature in psychology (Frieman, 2002; Chance, 2009) generally underscores three high-level steps: an agent must perform a behavior; a modification must follow this action (the phenomenon); the consequence of the modification must reinforce the action positively or negatively. As a result, the change in the action, situation, or environment can either strengthens or weakens the behavior. The change could also affect the form, range, frequency, persistence, timing, and magnitude of the behavioral response. In addition, the OC procedures generally includes recurrent cycles of spontaneous, voluntary or random actions followed by a reinforcement stimulus, whether it is removed or presented, rewarding or punishing and resulting in higher or lower reproduction of the contributive behavior. Finally, the behaviors could happen in chain and in a non-stationary environment, which dramatically increases the temporal order and complexity of the phenomenon (Touretzky and Saksida, 1997).

It has already been demonstrated that OC requires a different internal molecular mechanism than CC (Lorenzetti et al., 2006). Nevertheless, the latter seems to be involved in OC (Holland, 1993), reflecting the basic terminology of convergence and divergence where CC refers to stimuli-reinforcer (CS-US) and OC to a response-reinforcer (Behavior-Reinforcer) contingency. A comparative study (Baxter and Byrne, 2006) of these associative processes indicates that the convergence of the neural activity and the reinforcer in OC lead to a change in the neural membrane properties (modification of the input resistance and burst threshold), while CC is concerned with the modulation of a tagged synaptic site (Izhikevich, 2007). Also, several lines of evidence point to the dopamine as the excitatory neurotransmitter involved in the OC reinforcement process (Bédécarrats et al., 2013). Researchers in the domain are currently exploring some interesting molecular tracks (Lorenzetti et al., 2008) at the resolution of an analog single cell model (Brembs et al., 2002) to confirm which and how the components serves as the coincidental detector.

Computationally modeling the OC process at different conceptual levels depends on the desired outcome. The idea of developing an OC model using a rate-coded ANN is not new (Grossberg, 1971), nor is the idea to embed this learning process in a virtual robot (Graham et al., 1994) or a real one (Gaudiano and Chang, 1997). These OC models share a common goal: Apply the comprehension of the natural learning mechanism for the benefit of physical robots. For example, Gaudiano AND Chang demonstrate how real robots could learn from a modified version of Grossberg's CC and OC model in an unknown environment, a simple navigation task of avoiding or approaching from rewarding or punishing cues. These opposing behaviors match different sensory cues. Perhaps, one limitation of that article is that a robot could eventually learn approaching walls or any objects to receive a reward or, inversely, avoid light when punished. Still, based on rated-coded ANNs, their model sustains the generalization of environments and minimizes the tuning parameters for an egocentric robotic framework. Since timing of events is an important factor in OC procedures, ASNN is preferred.

On the other hand, attempts to develop a time-coded ASNN as basis for an OC model embedded in a bio-inspired robotic platform is more recent (Arena et al., 2009; Helgadóttir et al., 2013; Soltoggio et al., 2013) and merits further development. Our contribution consists in a simple computational OC model, built with an elementary micro-circuit that can be generalized and applied in multiples OC scenarios, demonstrating that this learning process could involve only a small number of specific cellular elements. More precisely, an input feeding Cue neuron, an Action neuron and a Predictor neuron receiving a reward or punishment seem sufficient to compose the core of OC when connected by specific directed links and few learning rules such as habituation and STDP.

We base our approach on the fact that the OC phenomenon is seen in the Aplysia, and also in smaller neural organisms such as *C. elegans* (300 fixed neurons), which are already known for non-associative and associative learning (Qin and Wheeler, 2007) for adaptive behaviors. Moreover, this flat worm is perceived to also exhibit a derived dopamine implication in many behavioral modulation roles (Vidal-Gadea and Pierce-Shimomura, 2012). As such, the low neural count in these organisms and their multiple adaptive learning capabilities may imply that a simple integrative OC model may be sufficient to reproduce them. However, until now, few computational models are integrating such bio-inspired neural knowledge for robotic training purposes and general AI behavioral applications. In this work, we embed an OC model in an ASNN acting as a robot brain, using learning functions such as habituation and STDP, and a few neurons and synaptic links. To reach the goal, the paper includes several OC procedures of incremental level of complexity, using four general categories of contextual OC: (1) Increased behavior with the expectation of receiving a positive reinforcer; (2) Increased behavior with the expectation of not receiving a negative reinforcer; (3) Decreased behavior with the expectation of not receiving a positive reinforcer; (4) Decreased behavior with the expectation of receiving a negative reinforcer.

Scenario A uses a simple contextual procedure to demonstrate the minimal components required to build an invariant OC kernel. It corresponds to the first item in the list above (bold). Scenario B switches to the opposite OC primitive polarity within the same learning experiment; in few time-steps online, the robot learns from the same sensory cue to avoid doing the action depending of the contextual type of reinforcer. In scenario C, we suggest a more elaborate and dynamical experiment by varying the parameters of the minimal OC components requirement. In scenario D, we embed a representative ASNN implementing an OC procedure in a real robot platform. In all four scenarios, the OC kernel remains unchanged.

To summarize, despite the fact that the exact mechanism of OC is still unknown, we propose in line with the current cellular level understanding of the natural process a simple OC model for virtual and physical robots by embedding a neural core within an ASNN framework. As novelty, we suggest that OC applied in general contexts could be understood in terms of an invariant minimal component requirement and show the benefit of integrating the habituation and the STDP learning rules into the kernel. Our intention is not to provide new theories of the natural phenomenon nor revising extensive features of OC. The scope of this OC model is limited to simple OC contexts, and remains to be tested in more complex situations. Nevertheless, we above all target generalizable functional outcomes in robots to further ground this important adaptive learning process into real world applications, beside natural intelligences.

The balance of the paper is structured as follows: In the next section, methodology, we describe the dynamics of the ASNN and the software used. The various tasks, scenarios and results are detailed in Section 3. Finally, a discussion and future work close this article.

## Methodology

**Figure 1:**
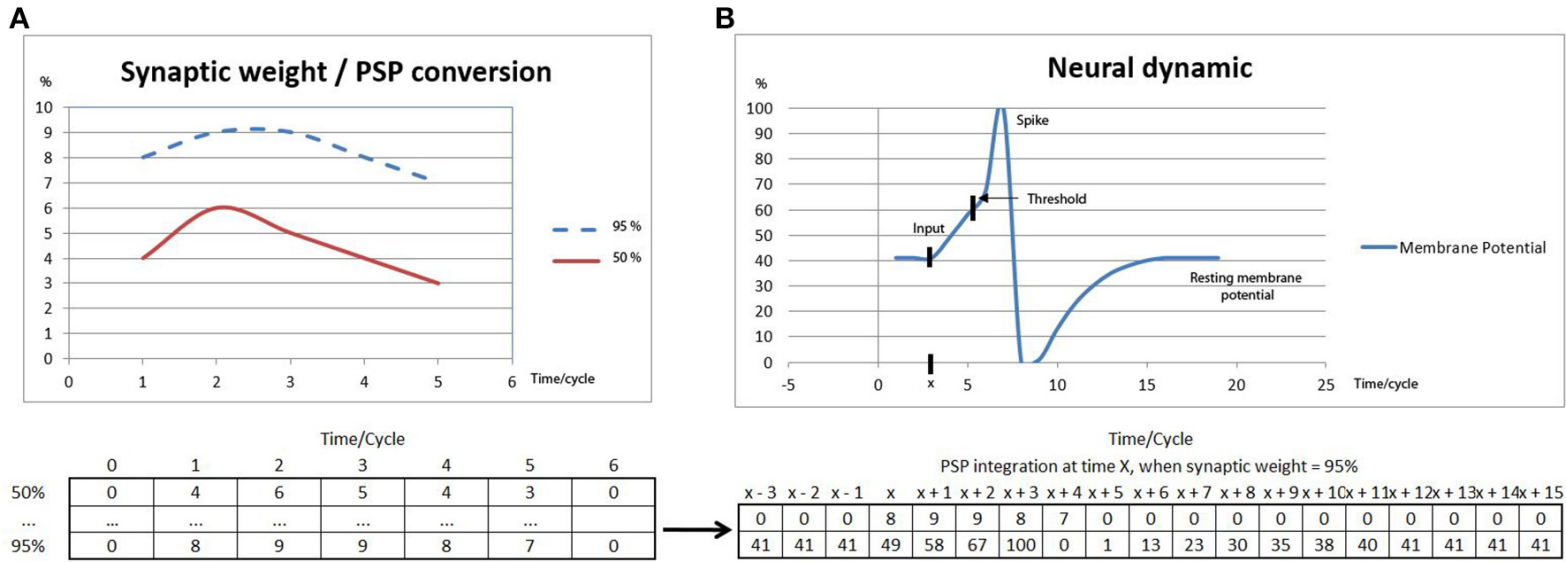
**(A)** Two different samples of synaptic weight values (see Table 2 in the Supplementary material) associated to PSPs, representing the membrane potential changes integrated on a 5 cycles time duration. **(B)** The membrane potential variation resulting from the integration of a PSP (weight = 95%) which starts at time *x*, allowing the neural dynamic to reach the threshold for a spike emission, after which a hyperpolarization state rapidly follows as well as the progressive return to the resting membrane potential value. Note, that at time *x* + 4, the PSP value is not integrated due to the absolute refractory period.

The OC procedures of this paper were implemented with the SIMCOG software, but other environments could have been used. In order to create a generic simulation environment, all the variables in SIMCOG related to the ASNN dynamics are normalized in the interval [0–100] and only integer values are used. With reference to Figure 1, a neuron is represented by an activation state whose membrane potential amplitude varies between 0 and 100, where 0 stands for the maximum of hyperpolarization, 63 is the threshold for firing and 100 the level of an emitted spike. The synapse between two neurons is represented by an excitatory or inhibitory link whose weight is a value varying also between 0 and 100. The different weights serve as indices in a vector table containing the sampled data of normalized PSP curves. Depending on the input and the current membrane potential state of a neural unit, a non-linear integration is performed across time, as done in a biological neuron, leading to a new membrane potential value and eventually triggering a spike emission if the firing threshold is reached. One feature of the ASNN editor in SIMCOG is the possibility to add sensory transducers to neurons. These unique components are intended to simulate the natural elements in the realm of robot sensors. Hence, these transducers form a bridge between physical or virtual world stimuli and their neural attachment, creating a sensory stimulus or graded receptor potential that serves as input to the ASNN. The sensory inputs also use a [0–100] scale, pointing to vectors that correspond to variations of membrane potential to receive. The same logic is applied for the motor neuron outputs, emitting sequences of current toward actuators, producing actions in virtual or physical world.

Lookup tables represent a strategy in computational modeling, including ASNN models (Ros et al., 2006; Alhawarat et al., 2013). Their use is motivated by the fact that neural models and variables are not always described by functions in closed form and, sometimes, experimental curves are the only precise data to rely on. In our work, we also used it to account for the lower CPU capacities in the robot's model. Notice that, since the curve shapes are similar for graded receptor potentials, PSPs and graded motor potentials, the same lookup tables is used (see Table 2 in the Supplementary material). A common fixed length of 5 cycles and a maximum change of 10% maintain coherence in the system regarding equivalent values in mV and ms of input/output and empirical results in the literature. Thus, the values that fill the vector table are set qualitatively, based on a progressive, non-linear stimulus-intensity scale. All values are integers in the range [0–100].

For all the neural branching parts (transducer/synapse/actuator) in the ASNN, different additive learning rules could be applied to obtain a desired modulation control (see Supplementary material, Equations 3–5). A sensory adaptation rule is used to restrict the data flow at the transducer site. An exponential decay factor is applied for a reduction percentage to occur on a short time period, thus allowing the model to deal with brief as well as constant stimuli. A habituation rule that acts between two neurons plays the same role essentially, but it filters redundant input information on longer and more variable temporal scales depending on the intensity, magnitude, frequency, and inter-stimulus interval of the input pattern. The habituation rule is a key factor in the OC dynamic we present, regulating the spikes traffic in the process of neural integration. Depending of the temporal input pattern, different exponential decay factors modulate the synaptic weights as well as recovery functions when the stimuli cease. Finally, we also applied a STDP learning rule at the synaptic site, for modification of the synaptic weight in relation of the precise pre-post spike timing (i.e., on a short time scale window, pre-post spike occurrences strengthen the link between two neurons and post-pre spike occurrences weaken it. All these learning rules are also implemented with integer values through lookup tables and built qualitatively from standard curve shapes in the literature.

**Figure 2:**
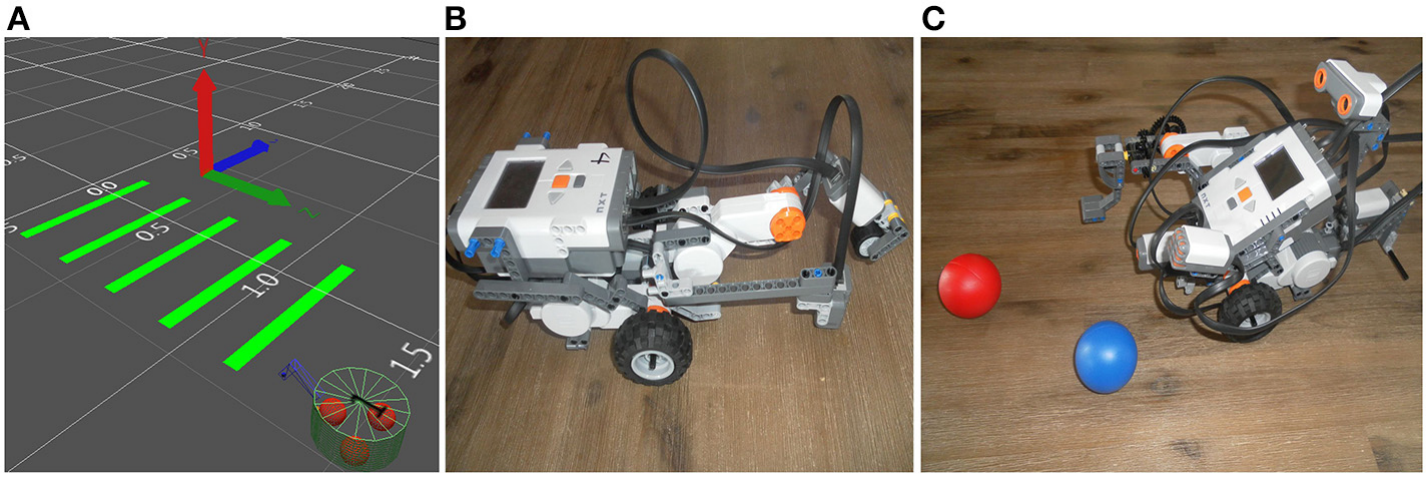
**(A)** Representative third person view of NeuroSim, the 3D-world simulator in the SIMCOG suite, showing a picture of the virtual robot when attempting to cross several green bars on the floor. **(B,C)** Similar structures with physical robots (Lego Mindstorms NXT 2.0).

In the virtual experiments, we choose a 15 cm diameter frame, two motorized-wheels at the back for locomotion and a support wheel at the front for balance (Figure 2A). A two-segment arm at the front of the virtual robot allows pressure on the ground such as color circles into complex scenarios. The physical robot is built using a Lego Mindstorms NXT 2.0 and intends to mimic the virtual one (Figures 2B,C).

**Figure 3:**
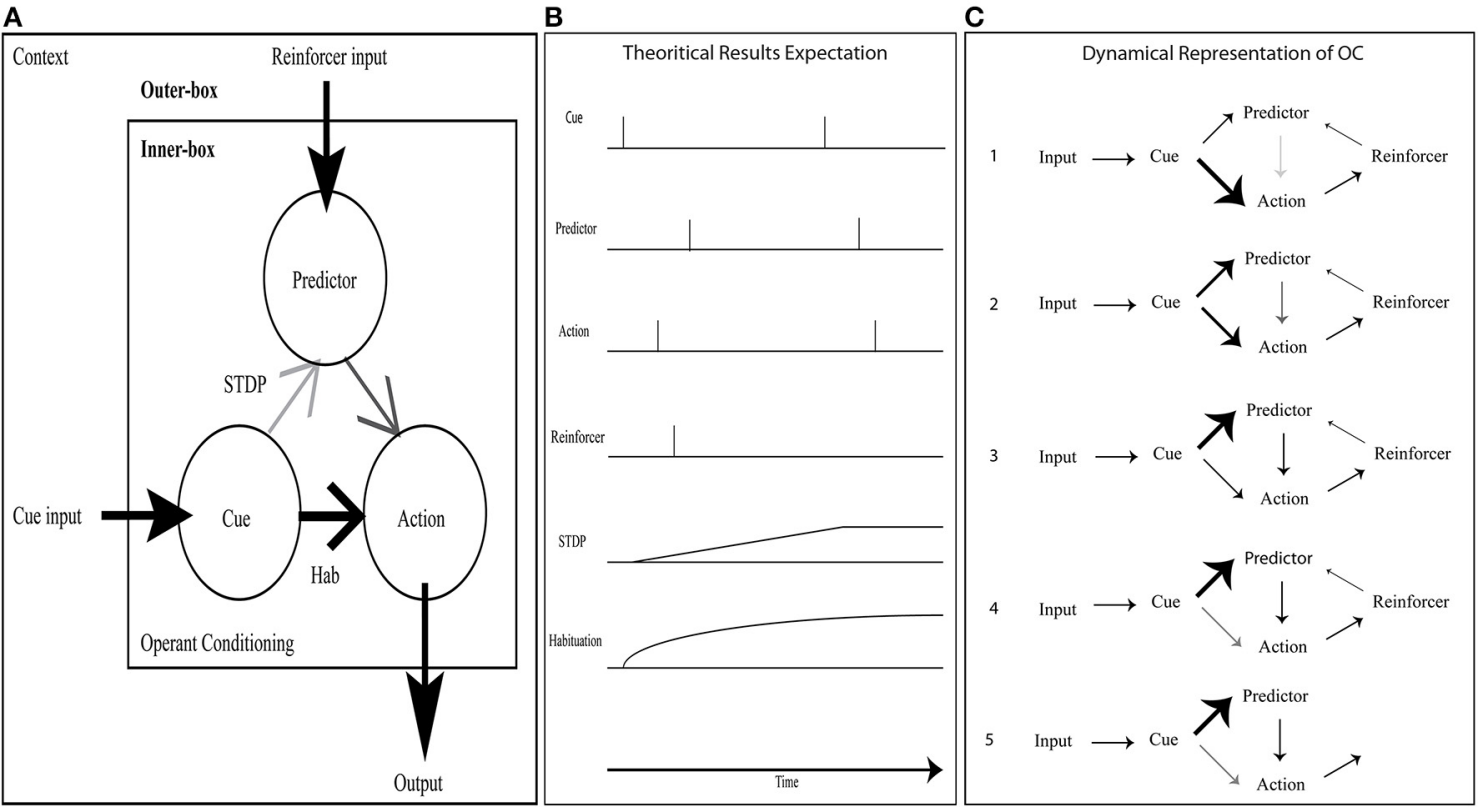
**OC simulation with a minimal neural component set. (A)** The inner-box consists of a Cue neuron feeding an Action neuron with a strong synaptic link (bold) and a Predictor neuron with a weak link (pale); the link between Predictor and Action is of intermediary strength. A habituation rule between Cue and Action allows decreasing the associated synaptic weight with time, leading to fade the Action. When a reinforcer input triggers a spike from Predictor, consecutively to a spike from Cue, the associated synaptic link increases due to STDP learning. It eventually allows Cue alone to stimulate Predictor and from this new path Action neuron. In the contextual outer-box, the input and output can be abstracted to any type of neural structure. **(B)** Dynamic representation of OC with the crucial components. Using a positive reinforcer (reward), the results are simulated through a timeline: Predictor leads to an Action without any Reward. **(C)** Dynamic step representation of the OC learning phenomenon with an emphasis on synaptic strength changes.

In the simplest OC procedure, the ASNN includes a kernel (inner-box in Figure 3A) built with three neurons, three synapses, one habituation rule, and one STDP synaptic rule. To accommodate the virtual and physical robots with a wide range of OC scenarios, the kernel is completed with a contextual outer-box that provides input from at least two transducers, output to one actuator, and three additional synapses that connect to the inner-box kernel (outer-box in Figure 3A). These last synapses come from the reinforcer input, the cue input and to the action output. We submit that this inner-box and outer-box combination has the minimum number of components to achieve OC procedures in varying contexts for a bio-inspired robotic paradigm (see Figures 3B,C for alternative dynamical representations of the OC model).

The contingencies of the OC procedures are tributary of the array of robot behaviors and the contextual environment, regardless of virtual and physical robot or natural agents. Still, the spontaneous production of behaviors is a necessary condition for any OC procedures. In all the virtual experiments that we conducted, the robots evolve in a closed 3D-world that contains different color bars painted on the floor, tactile sensors to detect the robot's arm pressing the floor, surrounding sound sensors to detect sound emissions from the robots and, vibration/light/heat emitters for cue input or rewarding/punishing reinforcers. Depending of the protocol, the robot's actions consist of emitting sound, moving, pressing the floor with an attached arm or pushing color bricks. Thus, the robot has restricted navigation skills and the a priori knowledge comes from the behavioral patterns inherited from the ASNN. The subsequent modulation of the responses comes uniquely from the learning rules, and the results depend on the temporal associations of the sensory cues, the reinforcers and the actions, which change with context.

## Tasks, scenarios, and results

Pecking a light or a color disk is a common task with pigeons in many learning studies. Pressing levers into a skinner-box or performing maze escapes are also stereotype experiments with rats, producing a vast literature in the learning domain. These apparently simple behaviors are more complex than they appear, and many variables manipulations are needed to observe the data on CC and OC procedures. In animal learning, the positive reinforcer usually consists in giving water or food when the animal is hungry or thirsty. In contrast, the negative reinforcer is often represented by brief electrical shocks, air puffs, or direct touches. The associative neutral stimuli used in natural contexts include flashes of light, sound, odor, color disks and mechanical devices (button, lever, and maze).

In this work, we abstract the behavior with sound emission in scenarios A and B. In scenario C, the action is to press on the floor and in scenario D to push blocks. The willingness to do or not an action can be resolved in many ways. Reflex action is a simple approach that we adopt here, though intrinsic neural properties could also lead to spontaneous endogenous random or periodic actions. Otherwise, any central pattern generators, value systems, or action selection mechanisms could also result in actions. In all experiments here, we explicitly propose an external reinforce, though again, alternative experiments could embed internal reinforcers such as a second ASNN standing for an intentional or motivational behavior. Scenarios A and B involve a positive reinforcer implemented as flashes of light and a negative reinforcer implemented as a vibration wave. In all scenarios, manual parameter adjustments were done. Efficiency was not a priority and more formal tuning methods could be used, such as genetic algorithms or other ANNs. Additional information on the parameter values used for scenario C is provided in the Supplementary material.

### Scenario A: primitive OC procedure (increased behavior/positive reinforcer)

**Figure 4:**
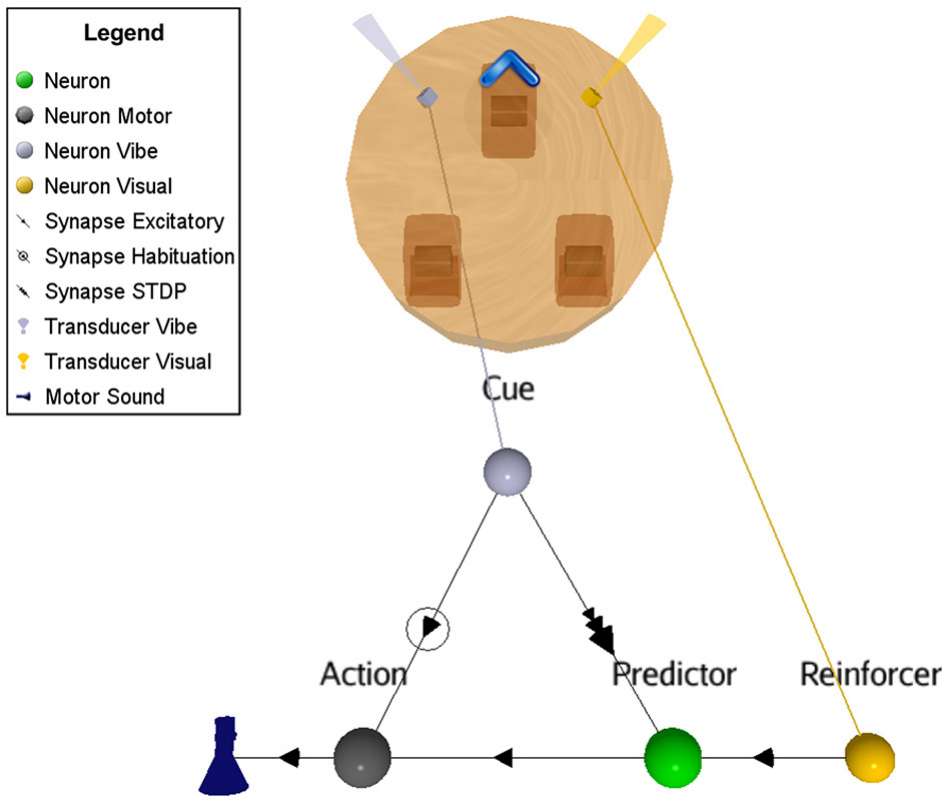
**Suggested ASNN to achieve OC procedures in varying contexts with a minimal component requirement**. This specific situation results in increased behavior when followed by a reinforcer. The positive reinforcer consists of light perceived from a visual sensory neuron. The action is simulated with a sound emission.

The ASNN associated with the robot configuration is shown in Figure 4. It allows realizing the primitive OC procedure of increasing (all or none in this experiment) the behavior when followed with a positive reinforcer. This basic configuration can be generalized to the other primitive cases and it represents the proposed minimal neural components requirement. In particular, swapping the excitatory synaptic link for an inhibitory one between the Action neuron and its actuator will result in decreasing the behavior. The attribution of a positive or negative aspect of the reinforcer corresponds to an internal value, which depends on the context. Hence, the four primitive OC scenarios could be embedded in the same structure.

**Figure 5:**
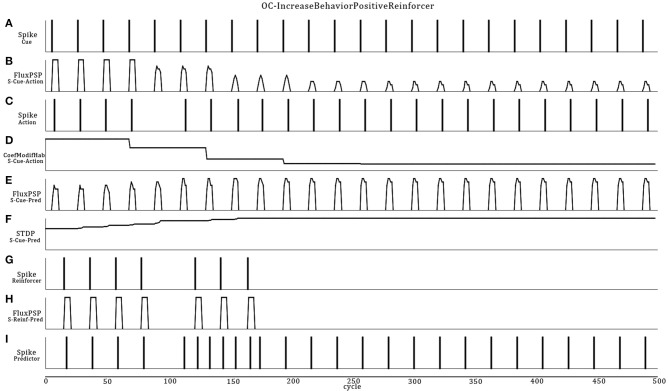
**Dynamic ASNN states (Figure 4) involved in an OC procedure**. The Cue neuron **(A)** emits spikes in reaction to regular vibes pacing into the virtual world. The strong synaptic link between Cue and Action first commands the robot to do actions **(C)** implemented as sound emissions. Because the synapse between Cue and Action incorporates a habituation rule which decrease the synaptic efficiency **(D)**, the PSP eventually fades enough to stop the action around cycle 100 **(B)**. Simultaneously, Cue emits spikes toward the Predictor neuron, causing small PSPs **(E)**. During the first 100 cycles, the synaptic weight **(F)** between Cue and Predictor is not strong enough to lead to a spike. The Reinforcer sensory neuron **(G)** spikes unconditionally from a light perception producing unvarying strong PSPs **(H)** toward the Predictor neuron **(I)**. The reward comes from the virtual world in response to the action. The spiking pattern of Predictor results from the integration of growing PSPs from Cue/STDP and the PSP of the Reinforcer. Stopping the reward still produces the desired behavior in an expectative manner due to OC.

Referencing to Figure 5, the Cue neuron reads periodic vibrations emitted from the virtual world. Consequently, Cue emits spikes to the Action neuron that triggers a reflex sound emission from the robot. Flashes of light (Reinforcer) are emitted into and from the virtual world (supervised procedure) consecutively to the robot's action for a certain period. In this ASNN configuration, the endpoint is to provoke a sound emission in the expectation of a rewarding light. At the synaptic link between Cue and Predictor, the STDP learning rule associates the sound emission (from the Action neuron) and the ensuing positive reinforcer (reward input) to the Predictor neuron. Discontinuing the reward (around cycle 175), reveals the ascending strength of this synaptic link, leading to predict alone the reward from the cue.

Four control situations can exist (not graphically shown): (1) Without the habituation rule, the action never stops and removes the benefit of predicting the reward, as well as producing the action without the possibility to stop it in the case of a negative reinforcer; (2) Without the STDP rule, the reward is always requested to produce the action and the cue will never be strong enough to predict the reward (3) If the reward precedes the action, the STDP rule will decrease the synaptic weight and the action stops rapidly, highlighting the non-predictive value of the link (4) Finally, stopping the cue will lead to “forget” the association, an extinction feature of OC procedures. We also did simulations on all learning rule permutations within the OC core and found no other possibility to produce OC.

The synaptic weights must be well balanced in order for the OC to work properly. Within the previous learning rules permutation tests, we randomized several times the initial synaptic weights and found no other concluding experiments. More precisely, we achieved the OC procedure only when there was a high synaptic weight between Cue and Action, and a low weight between Cue and Predictor.

### Scenario B: two opposite primitive OC procedures (increase behavior/positive reinforcer) then (decrease behavior/negative reinforcer)

**Figure 6:**
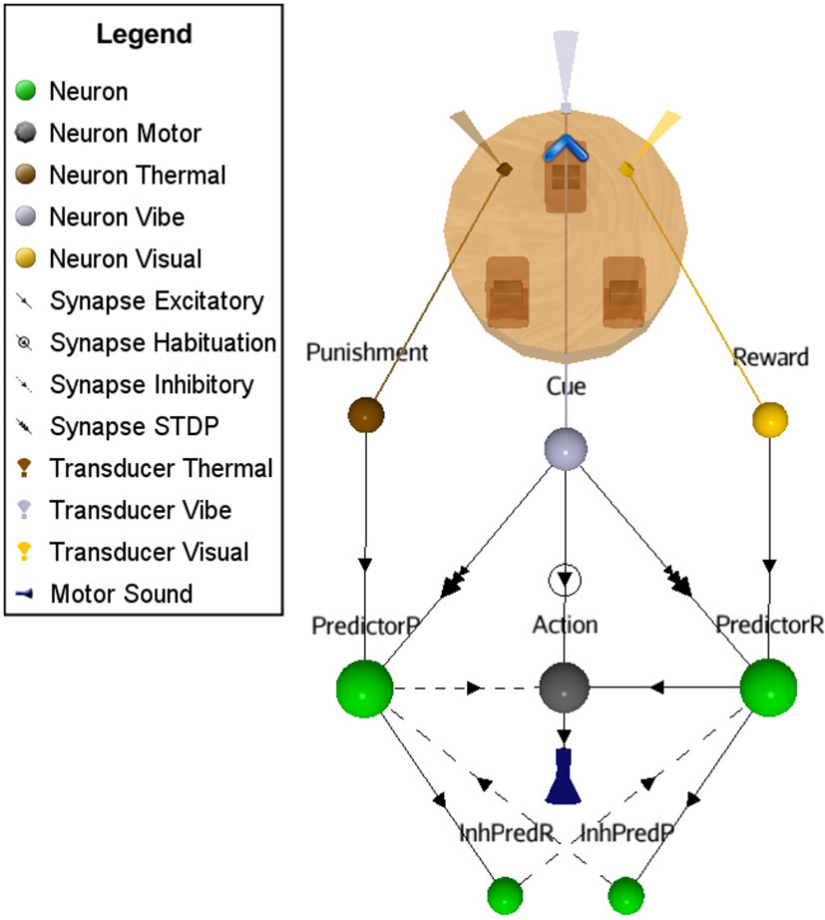
**ASNN and associated external robot structure built to exhibits opposing behaviors of emitting or not a sound**. The proposed ASNN includes the previous one (Figure 4), sharing some elements of the minimal components requirement. The Punishment sensory neuron (heat) is added as a negative reinforcer and renamed Reward neuron as in the positive reinforcer (light-visual). We individualized the Predictor neurons in respect of the attached reinforcer. Each behavior mutually inhibits the opposite one.

**Figure 7:**
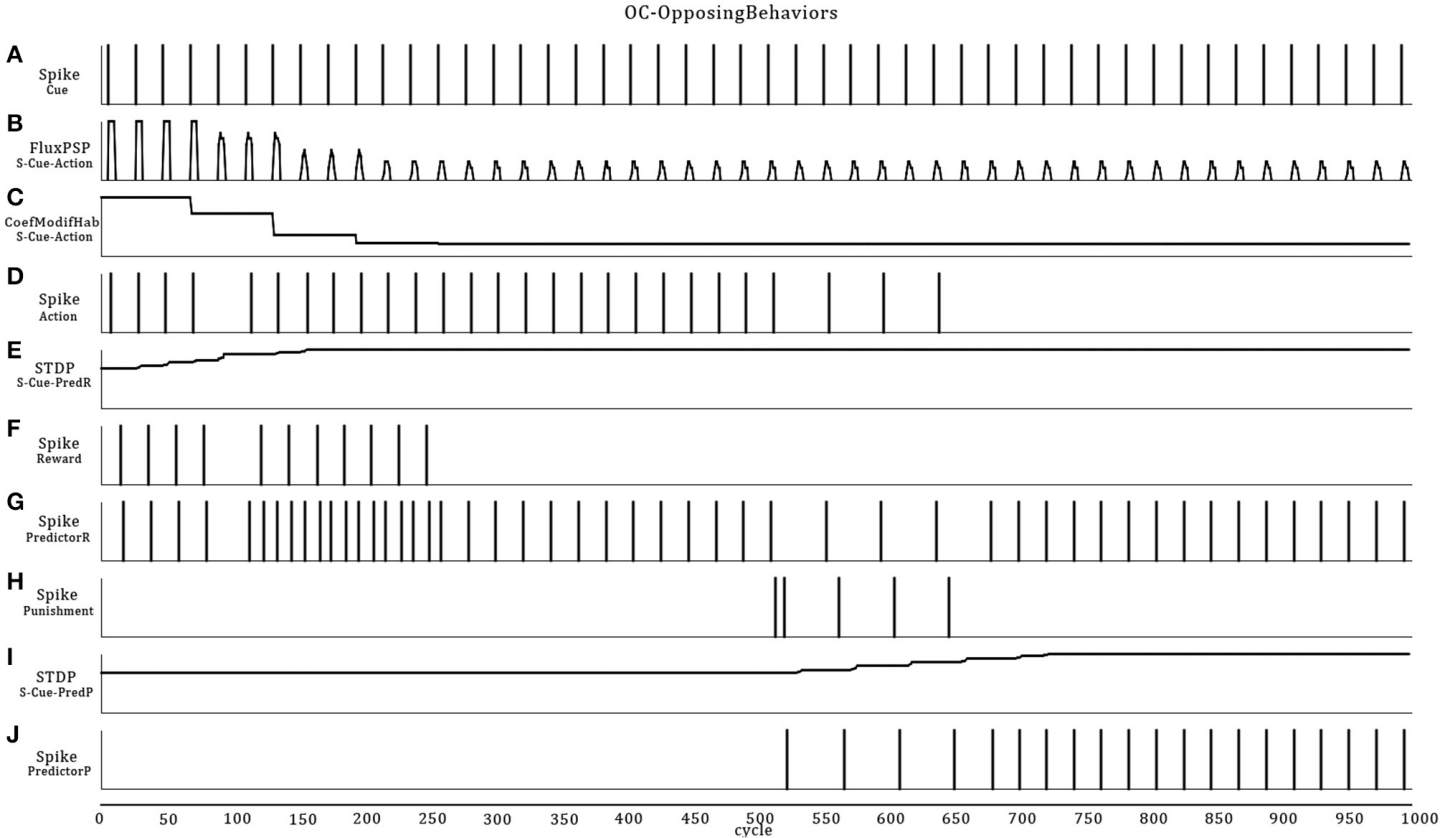
**Extension of the OC scenario A (Figures 4, 5) by following the response with a punishment**. The result is that the robot first learns to emit sound to get a rewarding light and then, ceases emission to avoid a punishing heat wave. Thus, Cue regularly catches vibes from the virtual world **(A)**. The synaptic link between Cue and Action conveys PSPs with decreasing strength in time **(B)** from the habituation rule **(C)**. Then, Action only spikes **(D)** when the temporal summation of all incoming PSPs is strong enough. Because Cue also emits to PredictorR, PSPs are produced in time but with increased amplitude from the STDP rule **(E)**. When the robot emits a sound, the virtual world responds for the first 250 cycles with a rewarding light **(F)**. Thus, Cue reaches Action from PredictorP **(G)** and elicits sounds with the expectation of getting a reward, even if the world ceases to offer it. At cycle 500, the world turns on a punishing heat wave **(H)** each time the robot emits a sound instead of a rewarding light. The STDP rule **(I)** at the synapse between Cue and PredictorP increases the synaptic weight until Cue triggers PredictorP **(J)** alone without punishment.

This scenario represents a small increment in the ASNN complexity (Figure 6). We kept the simple structure of scenario A, but add the possibility of punishment (negative reinforcer) with a brief heat wave. One can see the duplication of the minimal component structure. Note that these imbricates neural circuits could be used in separate OC procedures. In comparison to scenario A, some interneurons are added to link both circuits and explicitly highlight the mutual exclusive opposition of behaviors (sound on/off) and the effect of the reinforcer (heat/light). The neural dynamic of this OC procedure (Figure 7) leads to reverse the behavior of doing a sound to get the rewarding light by stopping the action from learning by punishment. This ASNN could be abstracted to any other actions like escape behaviors. One can see the persistence of the PredictorR spike pattern from the Cue input. In fact, the link is reinforced each time Cue spikes. When ceased (not shown), the STDP eventually enters in a recovery mode and simply forgets the association with a previous reward. The same logic could be applied with PredictorP.

### Scenario C: pressing color X in expectation of rewards or punishments

**Figure 8:**
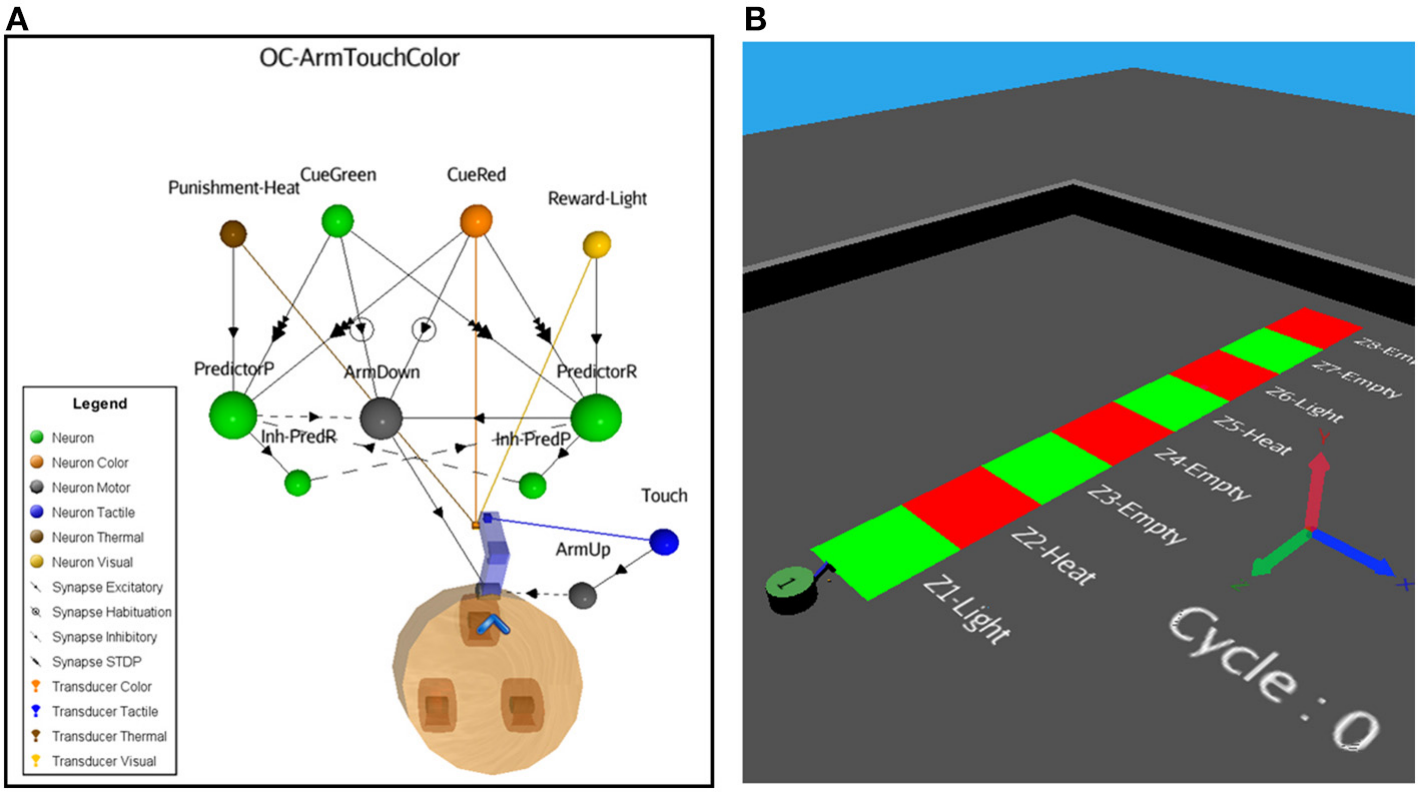
**(A)** ASNN corresponding to the arm-down behavior when the robot passes over color bars on the floor. The structure of a minimal components requirement is duplicated and shares a common Action neuron. **(B)** The NeuroSim virtual environment shows a robot that will move forward and experiments a stripe of different color zones on the floor associated to reward, punishment depending on the location.

In this more complex scenario, we keep the OC kernel untouched, but replace some external elements and add others. In Figure 8A, instead of a reflex sound response when the robot perceives a vibration stimulus, two new Cue neurons are introduced, now sensitive to color patches on the floor. Cue-Green is a sensory neuron responding to the green color and Cue-Red to the red color. Also, to encompass a larger type of input patterns, including constant stimuli, we add a fast adaptation learning rule (negative exponential decay factor with a fast recovery parameter) to the attached visual transducers. This biologically realistic function prevents an overflow of the input data. The color patches represent neutral stimuli. Thus, if seeing the colors persists, the sensory adaptation results in slowing down the spike emission.

In scenarios A and B, the robot was static, emitting a sound or not as simple behavior. Now, the robot moves forward at a constant speed and the Action neuron (Arm-Down) responds in moving down an attached arm when a specific green or red color on the floor is seen, until a gentle pressure on the color patch occurs. As a convenient accessory secondary behavior, every time the robot touch the floor, a sensory motor reflex (Arm-Up) replace the arm in its original position, determining a maximum rate of pressure on the floor. Because of the implemented habituation-learning rule between the Cues and Arm-Down neurons, the action will eventually stop if the stimuli are constant, preventing a looping and reflex situation if no significant events follow the behavior.

**Figure 9:**
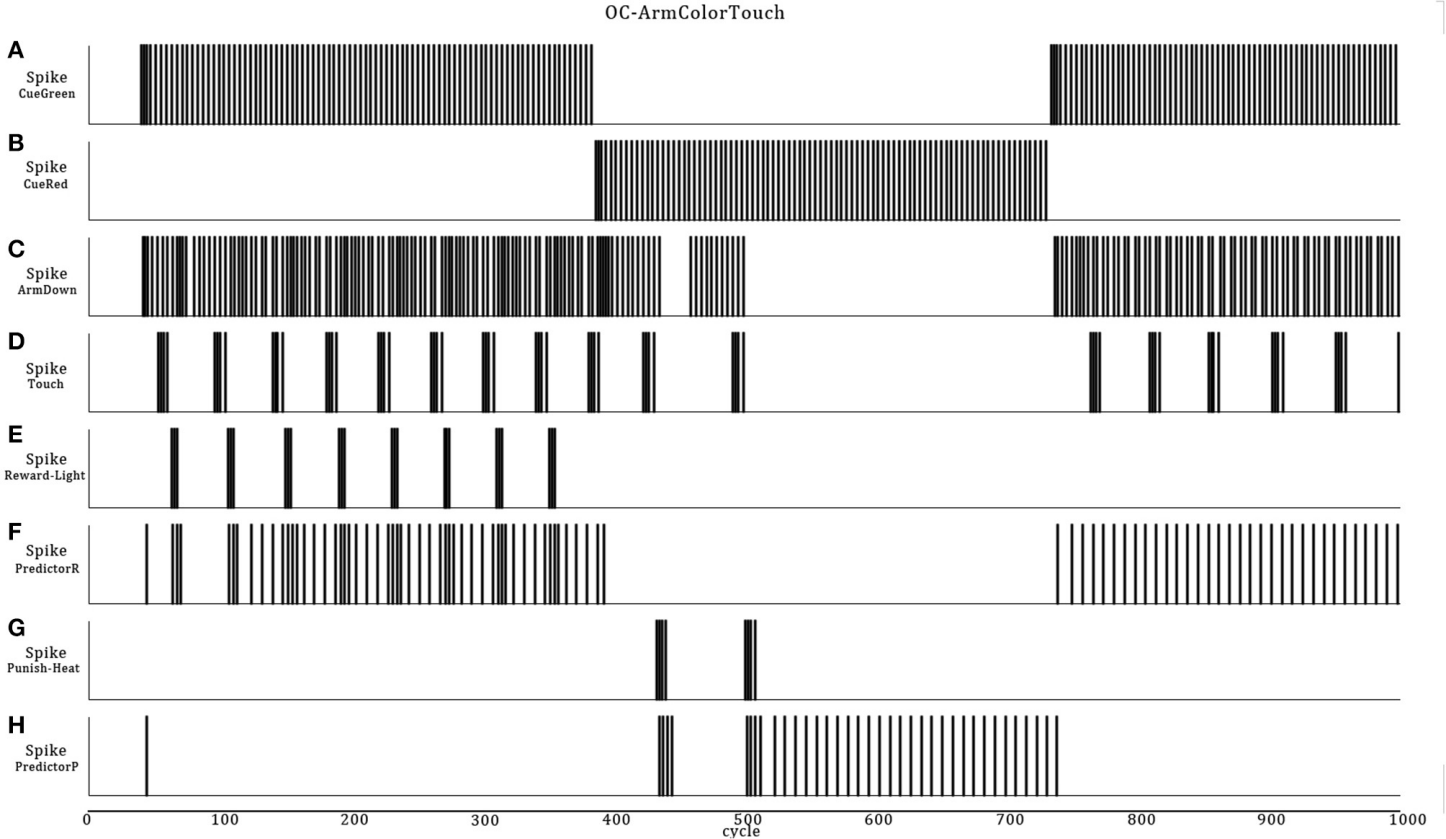
**Neural dynamics details of the OC-Arm-Down simulation when the robot passes through the first three zones**. The graphic shows the sensory color neurons **(A,B)** which are the Cues and leads to the Arm-Down behavior **(C)**, followed by touches on the floor **(D)**. These actions trigger automatically a rewarding light **(E)** or a punishing heat **(G)** from the 3D-world, depending of the zone and the color. The associated Predictor neuron eventually spikes to get or avoid a reward **(F)** or a punishment **(H)** without the reinforcer stimuli.

**Figure 10:**
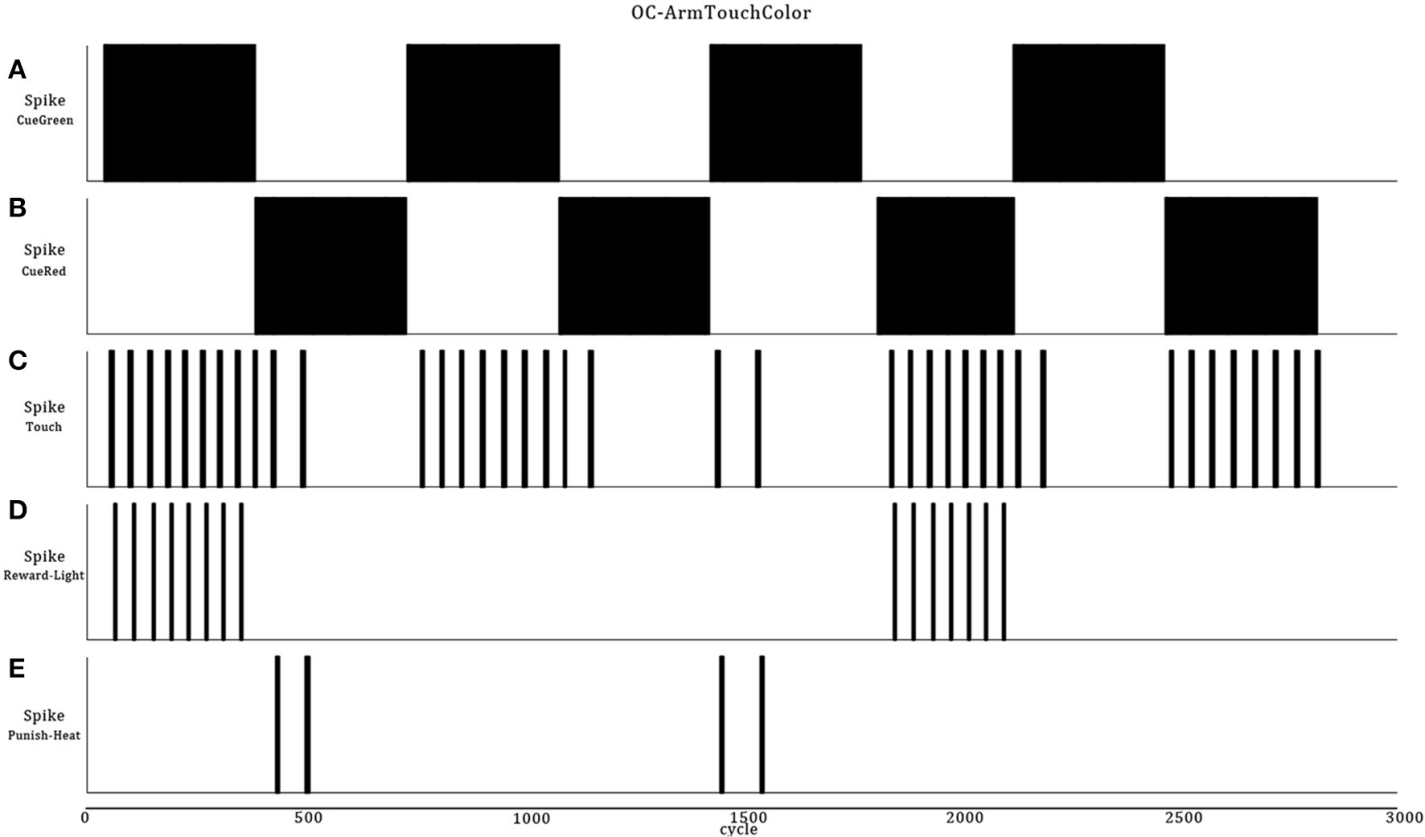
**Integral path of the robot when it passes over eight color zones (A,B)**. The data are the same as in Figure 9 but, the second half portion reverses the associated reward **(D)** or punishment **(E)** stimuli in regard of the color bars. At the end, the robot learns to avoid pressing the green bars and presses the red one **(C)**.

From these scenario elements, it is easy to introduce an OC procedure of any of the four primitive types. We choose to give a reward when the robot presses on the green bars (Increase-Behavior-Positive-Reinforcer) and a punishment (Decrease-Behavior-Negative-Reinforcer) when it presses on the red bars. The expected behaviors are that if the robot perceives a red or green color on the floor, it will presses on the floor with its arm for several times and then adapt from the habituation and sensory adaptation learning rules. Depending on the input stimuli pattern (Figure 8B), the adaptation and habituation or their recovery phases, when the color changes or ceases the robot may eventually respond again to the previously adapted color. After, if there is a reward (light) following the action of pressing a color, the robot overpass the adaptation/habituation learning rules and continues the pressing behavior, expecting a reward from the PredictorR neuron and the STDP rule that previously increased the synaptic weight. The same is true for the opposite situation, but the robot responds by preventing pressures if a punishment (heat) follows the Arm-Down movement. As in scenario B, the ASNN could afford a dynamical transition from the reward and punishment, but as a novelty, it could also adapt from the associated cue color stimuli (Figures 9, 10).

### Scenario D: physical robot aiming to discard wrong color pieces through an OC procedure

**Figure 11:**
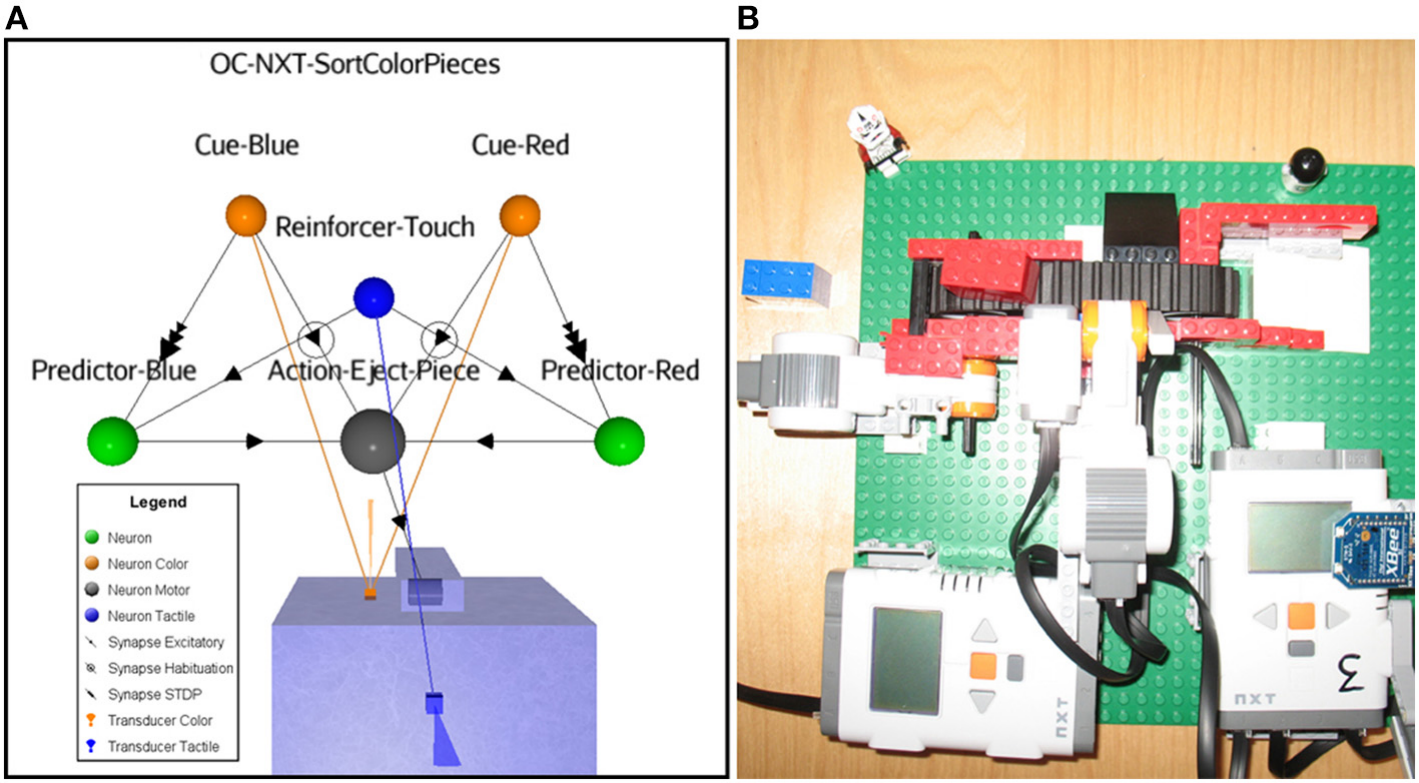
**(A)** ASNN of a robot sorting color pieces; it chooses between a red or blue one depending on an external touch reinforcer. **(B)** Conveyer belt with bricks and robot (No. 3) flipping from a half rotation movement with a side arm to eject the pieces. A color sensor located at the front of the robot allows it to reads the colors of the pieces. Also, a touch sensor is attached for external reinforcing.

In this last proof of concept scenario, we use a Lego Mindstorms NXT 2.0 robot to test the OC process implementation in a physical platform. Here, a static robot acts on a conveyer belt that transports blue and red color pieces (Cues). The action consists in first ejecting off the belt any perceived color pieces. Without reinforcement, the sensory adaptation and the habituation learning rules eventually lead to stop the behavior, the robot becoming non-selective for any color pieces. With the OC procedure, the goal consists at learning to selectively discard pieces according to their color, keeping the “good” ones on the conveyer belt. The rewarding reinforcer is implemented as a tactile input from a dedicated sensor. We show the associated ASNN (Figure 11A) and a picture of the corresponding physical robot (Figure 11B). For example, if the red pieces are reinforced by touches after they pass in front of the color sensor, eventually the blue pieces will always stay on the belt and the red pieces always be rejected (supplementary materials are available at http://www.aifuture.com). One can observe another configuration of the neuronal architecture including the minimal components requirement, sharing again portion of the critical elements with the Action and the Reinforcer neurons.

## Discussions and future works

Predicting the future gives clear advantages to natural organisms in terms of adaptation to survive. Learning the consequences of our own actions represents one of life's mechanisms, and it characterizes the OC process. In the real world, autonomous mobile robots must deal with uncertainty and need concrete and fast solutions for adaptation to different contingencies. Learning from OC procedures is certainly an avenue to consider for seeking “good” and avoiding “bad” situations.

In this paper, we showed through virtual robots involved in few OC scenarios of different level of complexity that a minimal component structure persists in the ASNN controller. We also validated the OC model in a physical robot. We suggest that this invariant kernel may be a sufficient condition to define the OC process in terms of cellular elements, including a STDP and a habituation synaptic learning rule. The first experiment, scenario A, point to a structure that could represent this elemental “brick” of the OC process when embedded in an ASNN. Our incremental simulation approach reveals that a variation of the contextual elements or copies of the minimal kernel requirement are all that is needed for the OC phenomenon to proceed.

The challenge in low-level explanations of the OC mechanism is to find the convergence of behavioral consequences and reinforcer. We have proposed a simple neural circuit pointing to clear elements at the cellular resolution level. The OC model seems general enough to maintain explanations in many contexts.

The power of OC learning is, in a given context, to shift dynamically a neutral spontaneous behavior toward a preferred behavior from the association between one action and its consequence. The context is often coming from sensory cues. The value of the outcomes must also be embedded somehow and should be part of the dynamical process. Persistence, recall, and reversibility are also essential plastic learning features that lead to learning adaptation. In scenarios B and C, we showed these modulated capacities from changing opposite behaviors online and from reversing the neutral type of initial cues. The conclusion is that a stimulus, a behavior or a consequence could be good one day and bad another day. Thus, a computational model of OC should be concerned by all these essential characteristics.

In scenario D, we demonstrated that the OC process was possible in a physical robot by transferring the ASNN into it. We also varied the neural architecture as well as the temporal aspects of some parameters. By randomizing the pieces order (Cues), we showed that the STDP rule could modulate the synaptic weights with different time intervals, as long as the ISI (inter spike interval) is included in the time-window of the STDP rule. Also, the presentation of the piece-cue-stimulus was time-varying, depending of the user that feeds the chain. Moreover, because the timing of the touch (Reinforcer) was also realized with a user (imprecise), it brings some other interesting temporal variation into the OC procedure. In a robotic perspective, sorting color items is easy with programing. Changing the color on the fly is also possible, but the system should previously have programmed the feature. With our OC model, a supervised procedure could reverse any color pieces (or any other object's features). We showed also that no prior knowledge is needed because all cues, actions, and reinforcer are considered neutral and could be changed at any time. Such flexibility could be useful in several applications in the AI domain.

In a general OC goal-directed action model, the Predictor acts by pointing the desired behavior among sets of actions by previously associating a given behavior to a given context that imply rewarding or punishing consequences. The transition of circumstantial action into a habit for expectancies represents one feature of the OC phenomenon. We find that this OC feature, as an experience-dependant plasticity process, seems realizable with a small ASNN which includes few, but crucial minimal components requirement.

As criticisms, the proposed OC including CC, habituation, and sensory adaptation models needs some examples (arbitrary) of stimuli-spike-action for the demonstration. We have not exhaustively explored the boundaries, nor defined the exact relationship between all the temporal parameters of the learning rules that are merged into the OC model. Thus, the limit values of the cue, action, and reinforcer remain to be tested further in terms of strength, rate, and ISI parameters to complete the computational model, even though it was not our primary goal to develop a formal OC model. Also, value systems should be implemented in the OC model (Krichmar and Röhrbein, 2013); particularly when considering that learning drives do not always comes as external reinforcers (Santucci et al., 2013).

In this paper, it was convenient to apply the reinforcer in the same temporal windows as the STDP, habituation, and sensory adaptation learning rules. The distal problem solving, credit assignment or simply put, how to link an input stimuli to a delayed reinforcer are highlighted by many researchers (Sutton and Barto, 1998; Izhikevich, 2007) and different theoretical models support the idea at the cellular level with as example, the tagging synapse hypothesis (Päpper et al., 2011). Moreover, in the fruit flies heat-box, OC procedures (Brembs, 2011) show that they learned in few minutes to avoid a specific punishing tube. Thus, longer temporal considerations manner and should be included for a complete OC model.

In a next model, we plan to include a neural trace component with a decay factor in association with a general feedback system. Extending the temporal window of the coincidence would then increase the “good” synaptic link. It was not a specific aim of this article to implement distant temporal gap within the OC process, though, to our knowledge, we don't see much obstacle foreseeing this temporal characteristic.

Also, introducing a dopamine-like reinforcer as a main excitatory input for rewarding signal seems a logical step inclusion in a future OC model if achieving higher cognitive processes is a target (Wang et al., 2011). Implementing a particular neuromodulator in an ASNN could translate into a different shape of the PSP with a higher magnitude peak and a shorter time window, offering similar dynamic features of the natural component. In the same line, adding cholinergic inhibitory inputs could increase the ASNN modulation possibilities as in biological conditions (Chubykin et al., 2013).

The current understanding of the OC process seeks the convergence of behavior and reward signal at the molecular level through the synergistic interaction between different types of mediators (i.e., calcium ion and calmodulin-sensitive adenylyl cyclase concentrations). At the end, the coincidence detector may be understood by only one molecule, inducing changes in the neural membrane properties, which produce CC and OC as distinctive associative learning processes (Brembs and Plendl, 2008). We clearly don't want to go that far in our present computational OC model though it is still an option. We prefer to focus on the functional and temporal aspects of the learning process for bio-inspired robotic purposes and concrete AI applications.

As extension of this work, several other behavioral alternatives should be implemented to validate this simple OC model in complex situations. As such, we intend to challenge this learning concept in decision-making property (Nargeot and Simmers, 2011) and its possible implication in the action selection mechanism. Adding chaotic feature to the ASNN through differential equations instead of lookup tables may find benefit in the richness of the behavioral response. Also, more complex robots should embed the OC model to appreciate the real scope of this computational learning rule. Overall, with this OC core model, building incremental complex dynamical scenarios taking advantage of habituation, STDP, and OC intrinsic characteristics may offer more than additive behavioral adaptation in neurorobotics applications.

Also, studying non-elemental forms of learning is far more difficult and characterize higher cognition in animals (Giurfa, 2007). Exploring shaping behaviors, collective-decision in heterogeneous cognitive abilities, negative pattern discrimination [learn to discriminate a binary compound stimulus and reinforce A and/or B but not AB, (A+, B+, AB-)], feature neutral discrimination (B+, AC+, AB-, C-) or mastering transitive inference rules as in A>B, B>C then A>C base on OC and CC processes may be a path to follow.

In conclusion, this article showed a computational OC model underscoring a minimal component requirement in terms of specific cellular elements as explanation of the learning process. This original OC model was presented in line with the current understanding of the neuroscience knowledge at the cellular level of comprehension of the mechanism. We applied this OC model in a specific bio-inspired robotic paradigm through an incremental, but simple level of complexity within the proposed scenarios. The true impact and limitations of this OC model remains to be determined in a wider spectrum of applications. Yet, the singular modularity of the minimal component requirement certainly opens the door to resolve interesting tasks by AI agents from this unique structure in the OC learning process.

### Conflict of interest statement

The authors declare that the research was conducted in the absence of any commercial or financial relationships that could be construed as a potential conflict of interest.
