# Supplementary material for: Operant conditioning: a minimal components requirement in artificial spiking neurons designed for bio-inspired robot's controller
Source: Front Neurorobot. 2014 Jul 25;8:21. doi: 10.3389/fnbot.2014.00021 (PMC4110879; doi:10.3389/fnbot.2014.00021)

**Appendix**

| **Equation 1:** Discrete-time neural input integration function  v_m_(k) = f_k_ ( Ʃ v_t_(k) + … ) with f_k_(x) = 1 if x >= 0  = 0 if x < 0  Where,  v_m_(k) = membrane potential at cycle k  v_t_(k) = sum of the transducer/synaptic input | **Graphic 1:** Corresponding normalized membrane potential fluctuation when used with the neural starting parameters in Table 1.  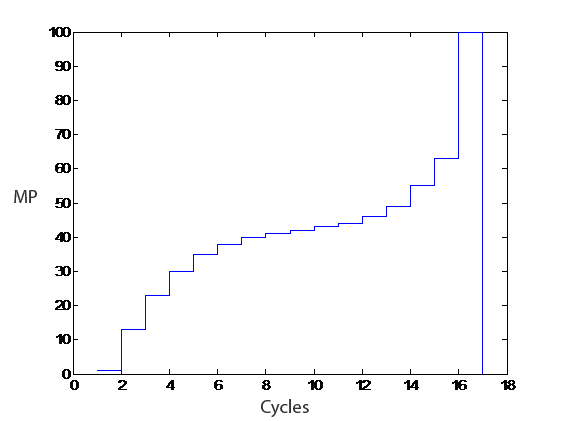 |
| --- | --- |

**Table 1:** General neural starting parameters proposed in a scalar percentage scale and relative time cycle


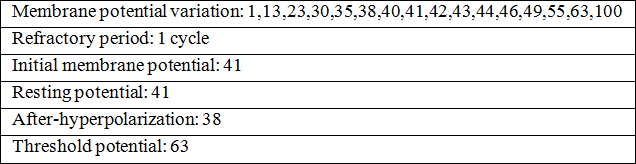


| **Equation 2:** General alpha function representing the descending part of the curve, which is similar to the production values of the lookup table.  f(t) = g * e ^(-t / tau)^  Where,  g = amplitude (10)  tau = exponential decay rate (~8)  t = time step (max = 5) | **Graphic 2:** General and complete theoretical curve shape of an alpha function representing a typical post-synaptic potential variation register at a post-synaptic element and depend of the current membrane potential and the nonlinear input summation at the current time cycle.  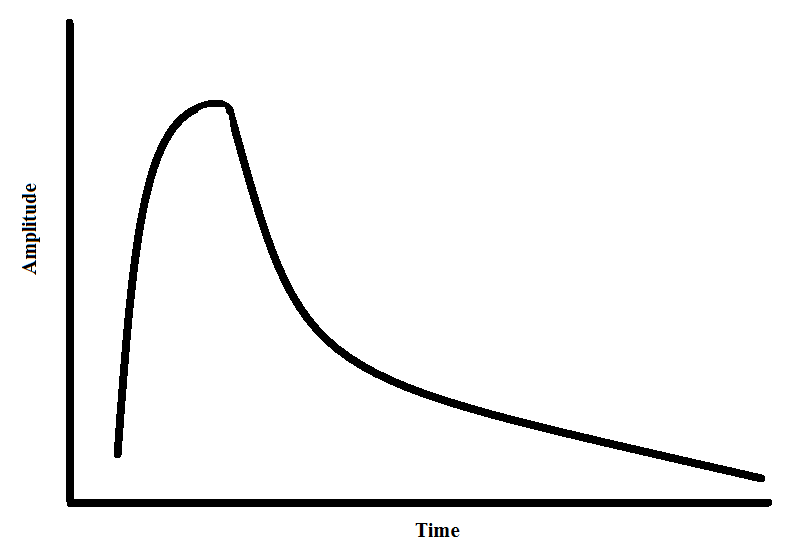 |
| --- | --- |

**Table 2: Lookup table used for the numerical percentage numbers of post-synaptic and graded receptor potentials. Each entry corresponds to a specific temporal sequence of a membrane potential variation to receive at the neural element depending of the stimulus strength**


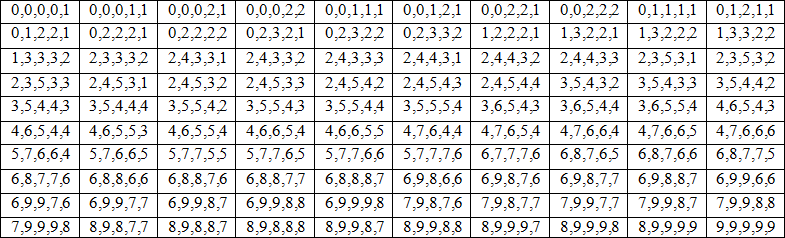


| **Equation 3:** General sensory adaptation function which inspired our adaptation rule values in the lookup table.  f(t) = ½ * k * ln(1+λ/t), t > 0  Where,  k = maximum amplitude (~8)  λ = decay rate (~50)  t = time | **Graphic 3:** General and theoretical curve shape of a sensory adaptation function involving a relationship between the amplitude/constancy of an applied stimulus and the relative efficiency through time.  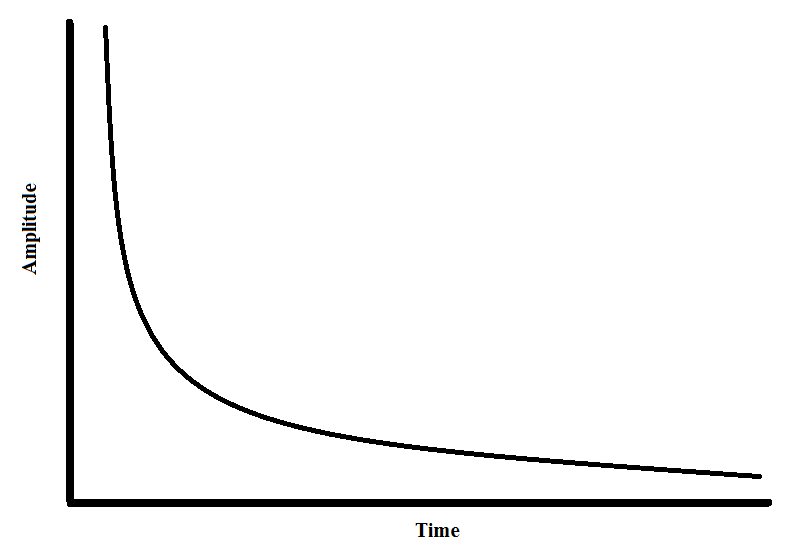 |
| --- | --- |

**Table 3:** Similar to qualitative results obtain from equation 3 and graphic 3, these are the simple numerical percentage values used in the sensory adaptation coefficient for the synaptic weight modifications when stimuli are constant. As an example here, the function reached the maximum of sensory adaptation in five time-steps which is fixed to 25%, with a 50% of decrement between each interval, considering a constant stimulus. If the stimulus cease at the transducer site, then a recovery phase reverse the coefficient through the same percentage at the same time interval.

| 25 | 12 | 6 | 3 | 1 |
| --- | --- | --- | --- | --- |

| **Equation 4:** General habituation function which inspired our habituation rule values in the lookup table.  f(t) = a * e ^(-b * t)^  Where,  a = initial pre-stimulus state (~100)  b = decay time (~0.3)  t = time  The recovery part is also exponential and follows a first-order positive step response | **Graphic 4:** General theoretical curve shape of the habituation and recovery phases when stimuli are applied and then ceased. The graphic represents a negative exponential growth function that is followed by a positive exponential growth function (which represents the recovery part). Different similar curves could numerically be resolved to obtain a variation of the habituation and recovery coefficients affection the synaptic weights variation in relationship to different ISI and strength parameters of the stimuli.  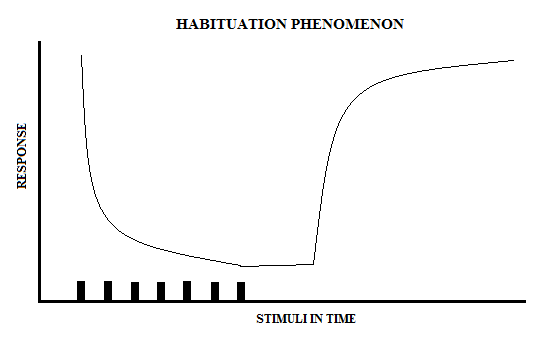 |
| --- | --- |

**Table 4: Example of a produced matrix in percentage numbers for a habituation rule**


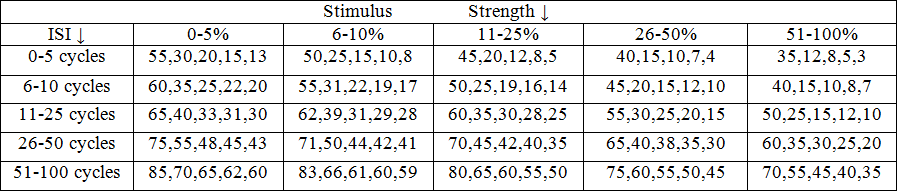


| **Equation 5:** Simple STDP function in additive mode which inspired our STDP rule values in the lookup table.  Δw = α _tpost – tpre_ e ^(tpost – tpre) / t^  Where,  α _tpost – tpre_ = constant (1 or -1) depending on the sign  of t_post_ – t_pre_  Δw = synaptic weight change  t = time | **Graphic 5:** General theoretical curve shape obtained from a STDP function, allowing the production of a synaptic modification coefficient. As an example, the maximal temporal window could be set to 100 cycles between the pre-post-spikes time arrivals. The maximum synaptic change could be set to 25%, occurring on five cycle duration with a nonlinear decay between each time step.  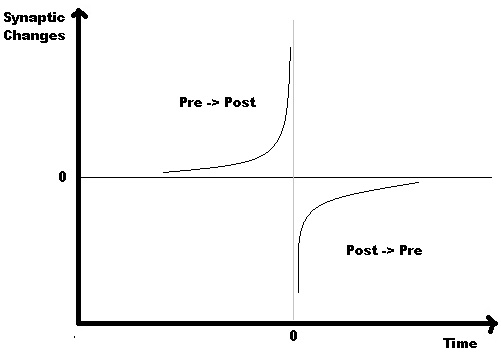 |
| --- | --- |

| **Table 5: Initial neural parameter values used in scenario C**  **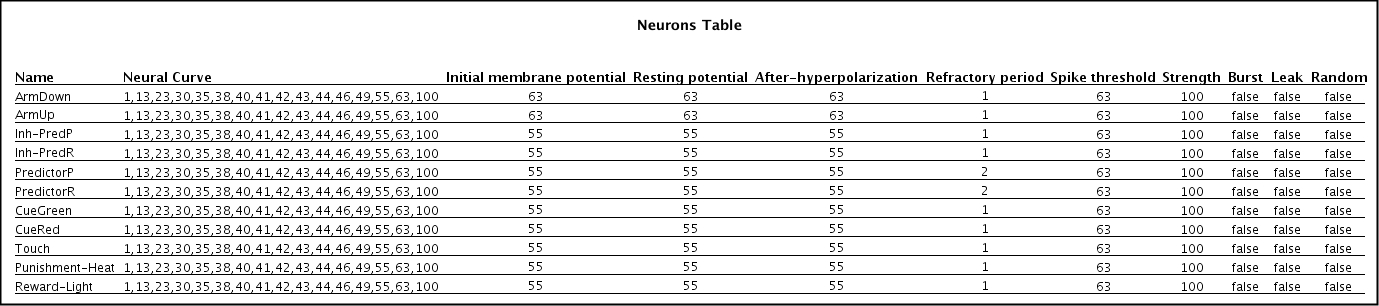** | |
| --- | --- |
| **Table 6: Initial synaptic weight values used in scenario C**  **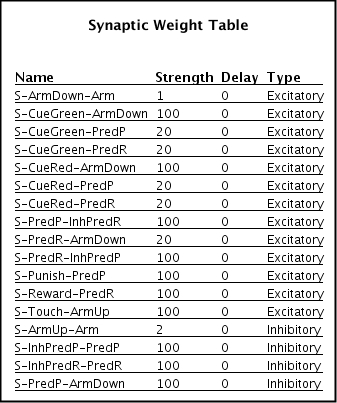** | **Table 7: Initial habituation learning rule parameter values used in scenario C**  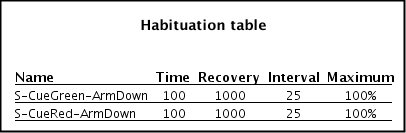 |
| **Table 8: Initial transducer sensory adaptation learning rule parameter values used in scenario C**  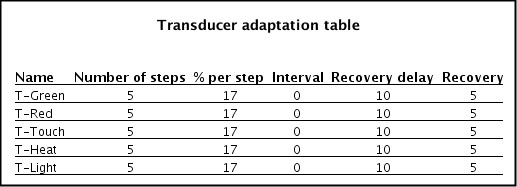 | |

**Table 9: Initial STDP learning rule parameter values used in scenario C**


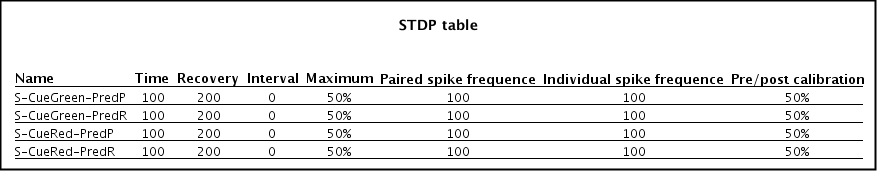

Supplement: Supplementary file 1 [file DataSheet1.DOCX]
